# Selective Extraction of Aromatics from Slurry Oil with Subcritical Water

**DOI:** 10.3390/molecules30092079

**Published:** 2025-05-07

**Authors:** Nuo-Xin Zhou, Zhu-Qi Liu, Meng-Han Zhu, Zi-Bin Huang, Jing-Yi Yang, Li-Tao Wang, Pei-Qing Yuan

**Affiliations:** 1 State Key Laboratory of Chemical Engineering, East China University of Science and Technology, Shanghai 200237, China; znx20002022@163.com (N.-X.Z.); liuzhuqi2000@126.com (Z.-Q.L.); zhumenghan_chem@163.com (M.-H.Z.); zbhuang@ecust.edu.cn (Z.-B.H.); jyyang@ecust.edu.cn (J.-Y.Y.); 2 PetroChina Petrochemical Research Institute, China Petroleum & Natural Gas Co., Ltd., China Petroleum Innovation Base, Beijing 102206, China

**Keywords:** slurry oil, aromatics, extraction, subcritical water, principal component analysis, Hansen solubility parameters

## Abstract

The selective separation of aromatics from slurry oil (SLO)—a low-value byproduct of fluid catalytic cracking—remains a major industrial challenge. This study investigates the use of subcritical water (Sub-CW) as a green and tunable solvent to extract aromatics from SLO in a semi-batch system operating at 250–325 °C. At 325 °C and a water-to-oil mass ratio of 6:1, the extract yield reaches 16 wt%, with aromatic hydrocarbons accounting for over 90 wt% of the extract, predominantly composed of 3- to 4-ring polycyclic aromatic hydrocarbons. Comprehensive characterization via simulated distillation, SARA analysis, FT-IR, and ^1^H-NMR confirms the selective enrichment of aromatics and effective separation from saturates and asphaltenes. To elucidate the molecular basis of this selectivity, principal component analysis of Hansen solubility parameters was performed. The results revealed a temperature-dependent solubility trend in Sub-CW, whereby the affinity for hydrocarbons follows the order aromatics > cycloalkanes > alkanes. This solubility preference, supported by both experimental data and theoretical analysis, offers new insight into subcritical solvent design and provides a basis for process intensification in SLO valorization.

## 1. Introduction

The crude oil processed by China’s refineries is generally heavy crude oil, and the production of light fuel oil is heavily dependent on secondary processing. Among them, fluidized catalytic cracking (FCC) contributes more than 50% of gasoline and diesel production. As the most important low-value-added by-product of the FCC unit, slurry oil (SLO) accounts for 3% to 7% of the raw material processed, with an annual output of more than 10 million tons [1]. SLO is rich in alkanes and aromatic hydrocarbons. Alkanes are excellent raw materials for FCC units, while aromatic hydrocarbons can be used as raw materials for value-added products such as rubber softeners and carbon materials [2,3,4,5].

The extraction of SLO using furfural as the main solvent has been widely studied. Zhang et al. used a composite solvent composed of furfural and n-octane to separate SLO [6]. The raffinate yield reached 76%, and the content of saturated hydrocarbons reached 75%. Gong et al. used furfural to separate SLO at different temperatures and solvent-to-oil ratios, finding that the H/C ratio and aromatic carbon content of each fraction were not much different [7]. The saturated hydrocarbon content of the raffinate reached 70%, while the aromatic hydrocarbon content of the extract reached 90%. Wang et al. explored the effect of additives on the extraction of SLO by furfural [8]. The experimental results showed that the application of composite solvents can improve the yield and quality of the extract. At 50 °C and a solvent-to-oil ratio of 0.5:1, the aromatic hydrocarbon content in the extract can be increased from 54.4 wt% of the raw material value to 82.9 wt%. However, the total content of resins and asphaltenes also increased from 5.1 wt% to 8.6 wt%. Wang et al. used a composite solvent composed of furfural and petroleum ether to extract SLO in a cross-current and countercurrent manner [9]. After three-stage extraction, the mass fraction of saturated hydrocarbons in the raffinate can reach more than 80%. Although furfural is recognized as an effective extraction solvent for SLO, it tends to resinify with aromatic hydrocarbons, causing coking and clogging of equipment [10]. In addition, furfural has adverse effects on human health and the environment. Therefore, the academic community has tried to apply other extractants like dimethylfomiamide and supercritical low-carbon alkanes (such as isobutane, butane, and pentane) to the separation of SLO [11,12,13,14].

Subcritical water (Sub-CW) exhibits thermodynamically tunable solvent properties, such as polarity, dielectric constant, and hydrogen-bonding capacity, which can be adjusted by varying temperature and pressure. For example, the dielectric constant of Sub-CW at 250 °C and 4.5 MPa is 27, comparable to that of methanol and ethanol, while at 325 °C and 13.0 MPa, it decreases to 17.0, approaching that of butanol. These features make Sub-CW a promising medium for extracting various classes of organic compounds. To date, Sub-CW has been explored for biomass valorization, remediation of contaminated soils, and enhanced recovery of heavy crude oil [15,16,17,18,19]. More recently, Zhu et al. applied Sub-CW to extract aromatic-rich fractions from vacuum residue (VR), achieving an aromatic content of 80 wt% in the extract and reducing asphaltene content from 15.5 wt% to 2 wt% [20]. These results suggest that Sub-CW holds significant potential for the selective extraction of aromatics from heavy oil fractions. However, the VR fraction studied in that work contained only 6 wt% saturates, limiting its representativeness for more compositionally balanced feedstocks such as SLO.

Despite these promising developments, key limitations remain unaddressed. Existing studies primarily focus on qualitative descriptions of Sub-CW extraction behavior or isolated component systems, lacking systematic elucidation of temperature-dependent selectivity or competitive solvation effects in multicomponent mixtures. Furthermore, thermodynamic models such as NRTL and UNIFAC, commonly used in Aspen Plus simulations, are designed for conventional solvents under near-ambient conditions and do not adequately capture the unique physicochemical behavior of Sub-CW, such as pressure-sensitive polarity, self-ionization, and hydrogen-bonding interactions. This often results in poor predictive accuracy for phase behavior and component partitioning under Sub-CW conditions. Consequently, there is a pressing need for experimentally validated, data-driven methodologies that integrate molecular characterization, solubility parameter analysis, and multivariate statistical modeling to guide solvent design and process optimization in Sub-CW extraction systems.

Herein, the extraction of SLO by Sub-CW was investigated in a semi-batch apparatus. First, the effects of water-to-oil ratio, extraction temperature, and extraction pressure on the extract yield were investigated. Then, various characterizations of the extract, such as group composition, boiling point, and gas chromatography–mass spectrometry (GC-MS) analysis, were performed to confirm the enrichment of aromatics in the extract and the effective separation between saturates and aromatics. The characteristics of applying Sub-CW in the extraction of SLO were further discussed by principal component analysis (PCA) of Hansen solubility parameters (HSPs) of hydrocarbons and Sub-CW.

## 2. Experimental

A SLO sample provided by China Petroleum & Natural Gas Co., Ltd. (Beijing, China) was used in the experiment, and its basic properties are listed in Table 1. The SLO used exhibits a high content of aromatics (67.6 wt%) along with a notably significant proportion of saturates (27.0 wt%). Sub-CW was used as an extractant, and its temperature varied between 250 °C and 325 °C. The pressure of the extraction system was 0.5 MPa higher than the saturated vapor pressure of water to ensure that the water was in a subcritical state. Some key thermodynamic properties of Sub-CW under the experimental conditions are summarized in Table S1 of the Supplementary Materials.

**Figure 1:**
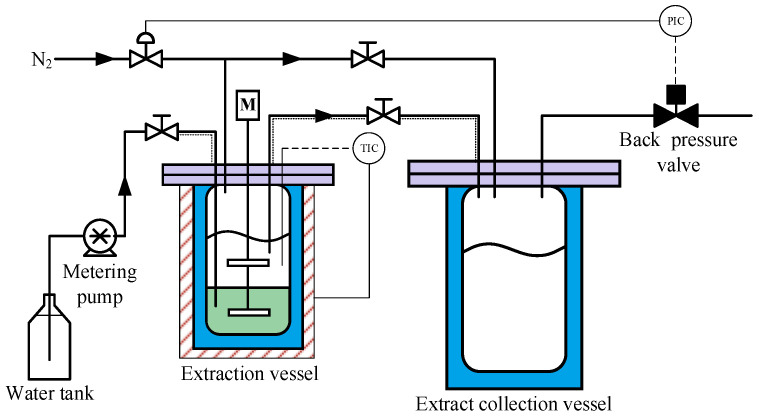
Apparatus for the extraction of SLO by Sub-CW.

The extraction was conducted in a semi-batch apparatus, as shown in Figure 1. The setup consisted of a 0.2 L extraction vessel equipped with a heating jacket, a 2 L extract collection vessel, a nitrogen pressurization system, a metering pump, and a back-pressure control system. The overall apparatus configuration and extraction procedure were similar to those reported by Zhu et al. in their study on Sub-CW extraction of vacuum residue [20].

A typical extraction process is as follows:

1. First, 100 g of SLO and 100 g of deionized water are loaded into the extraction vessel.
2. The extraction system is purged with low-pressure high-purity nitrogen (>99.999 vol%) and then pressurized with high-pressure nitrogen to the preset extraction pressure of 5 MPa to 13 MPa.
3. The extraction vessel is heated to the preset extraction temperature of 250 °C to 325 °C. Meanwhile, a stirring rate of 60 rpm is used to achieve limited mixing between the lower SLO phase and the upper water phase.
4. Amounts of 200 mL to 600 mL of deionized water are injected into the SLO phase using a metering pump. To prevent disturbance at the oil/water interface, the water injection rate is controlled at 2 mL/min.
5. Driven by the pressure difference, part of the water dissolved with oil components flows into the extract collection vessel.

The extraction under the same conditions is repeated three times to evaluate the effect of experimental error on the extraction performance.

The yield of the extract (*Y*_extr_) was calculated using

(1) $$Y_{extr}=\frac{m_{extr}}{m_{SLO}}\times 100\%$$

where *m*_SLO_ and *m*_extr_ represent the weight of the loaded SLO and the collected extract, respectively.

The extraction rate (*R*_comp_) of a specific oil fraction is defined as follows. The oil fraction can be one of the SARA fractions defined by group composition, one of the light fraction and VR fraction defined by boiling point, or one of the polycyclic aromatic hydrocarbons (PAHs) classified by the number of aromatic rings, etc.

(2) $$R_{comp}=\frac{C_{comp}Y_{extr}}{C_{comp,ini}}\times 100\%$$

where *C*_comp_ and *C*_comp,ini_ are the content of the oil fraction in the extract and the initial content of the oil fraction in SLO, respectively.

Following the standard of NB/SH/T 0509-2010 [21], the oil samples, including SLO and the extracts, were separated into SARA components, that is, saturates, aromatics, resins, and n-heptane insoluble asphaltenes [22]. Simulated distillation analysis of the oil samples was performed on an Agilent (Santa Clara, CA, USA) 7890B gas chromatograph according to the standard of ASTM D7169 [23]. Based on boiling point, the oil samples were classified into light fraction (IBP-500 °C) and VR fraction (>500 °C). The composition of the oil samples was analyzed and quantified on a Thermo Scientific (Waltham, MA, USA) ISQ719020 GC-MS system, following the standard of SH/T 0659 [24]. Hydrogen nuclear magnetic resonance (^1^H-NMR) analysis of the oil samples was carried out on a Bruker (Billerica, MA, USA) Avance 400M III superconducting Fourier transform NMR spectrometer. Tetramethylsilane and deuterated chloroform were used as the internal standard and the solvent, respectively. Fourier transform infrared spectroscopy (FT-IR) analysis of the oil samples was conducted on a Nicolet (Madison, WI, USA) iS10 infrared spectrometer.

## 3. Results and Discussion

### 3.1. Effect of Operating Conditions on Extraction Performance

**Figure 2:**
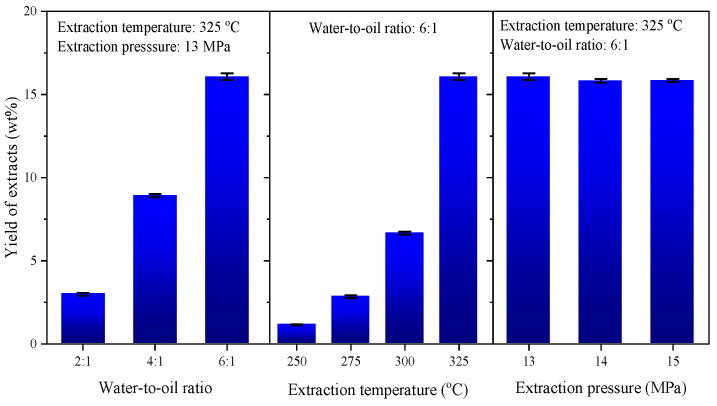
Effect of operating conditions on the yield of extracts.

According to literature reports, heavy oil components do not undergo chemical transformations within the experimental temperature range of 250–325 °C, as the cleavage of C-C bonds typically requires temperatures above 350 °C. Therefore, the compositional changes observed in this work can be attributed solely to physical extraction rather than chemical reactions [25,26]. The effects of water-to-oil ratio, extraction temperature, and extraction pressure on the yield of extracts were examined first, with the results shown in Figure 2.

The *Y*_extr_ increases significantly with the water-to-oil ratio at a fixed extraction temperature of 325 °C and pressure of 13.0 MPa. When the ratio increases from 2:1 to 6:1, *Y*_extr_ rises sharply from 3.0 wt% to 16.1 wt%, indicating that sufficient water content is critical for driving mass transfer across the oil–water interface. This trend suggests that a larger water phase volume facilitates improved solubilization and transport of extractable components into Sub-CW. It can be expected that an even higher *Y*_extr_ will be obtained along with increasing water-to-oil ratio.

Temperature also exerts a pronounced influence. At a fixed water-to-oil ratio of 6:1, *Y*_extr_ increases monotonically with temperature, reaching a maximum of 16.1 wt% at 325 °C. This behavior is attributed to the temperature-induced reduction in Sub-CW polarity, resulting from the disruption of hydrogen bonding networks between water molecules. The shift in solvent character promotes better compatibility with low-polarity hydrocarbons such as PAHs and saturates, enhancing their extraction. Notably, this yield surpasses that reported for VR under similar conditions (6.6 wt%), reflecting the greater extractability of SLO, which has a higher proportion of mid-boiling aromatics and fewer heavy, polar components [20].

In contrast, extraction pressure has only a minor influence on *Y*_extr_. When the system pressure varied from 13 to 15 MPa at 325 °C, the yield fluctuated only within ±0.3 wt%, despite a measurable increase in Sub-CW density from 658 to 665 kg/m^3^. This limited sensitivity can be attributed to the compressible nature of subcritical water, which allows its physical properties—such as density, viscosity, and dielectric constant—to change with pressure [27,28]. However, within the pressure range examined in this study, these variations are relatively modest and insufficient to significantly alter the solubility behavior of target components. As a result, the extraction process remains primarily governed by temperature-induced polarity shifts, rather than pressure-dependent density effects. This observation is in agreement with previous reports on the low-pressure sensitivity of Sub-CW extraction systems under comparable conditions [29].

**Figure 3:**
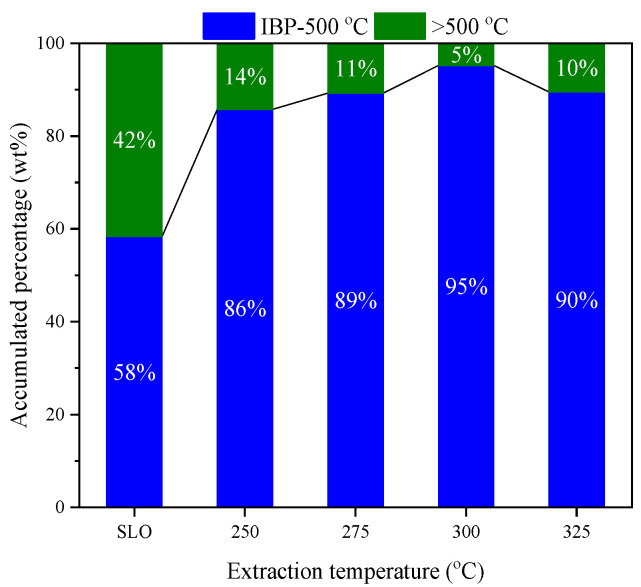
Boiling point distribution of SLO and extracts.

Figure 3 shows the boiling point distribution of the raw SLO and the extracts obtained at various temperatures. The original SLO consists of 58 wt% light fraction (boiling point below 500 °C) and 42 wt% VR (above 500 °C). Upon Sub-CW extraction at 250 °C, the proportion of light components in the extract increases markedly to 86 wt%, despite the overall extraction yield being relatively low (<3%). As the extraction temperature increases, the proportion of light components in the extract continues to rise, reaching a peak of 95 wt% at 300 °C. Even at 325 °C, where the total extraction yield reaches its maximum (16.1 wt%), the light fraction still dominates at 90 wt%, indicating that Sub-CW consistently favors the extraction of lower boiling components. This selective enrichment of the light fraction suggests that Sub-CW exhibits preferential solvation behavior toward mid-boiling and low-polarity compounds, which is consistent with its tunable polarity and reduced hydrogen bonding at elevated temperatures. In contrast, the extraction rate of the VR fraction remains low across all temperatures, likely due to the stronger intermolecular interactions and higher molecular weights associated with those compounds.

### 3.2. Selective Extraction of Aromatics from SLO

**Figure 4:**
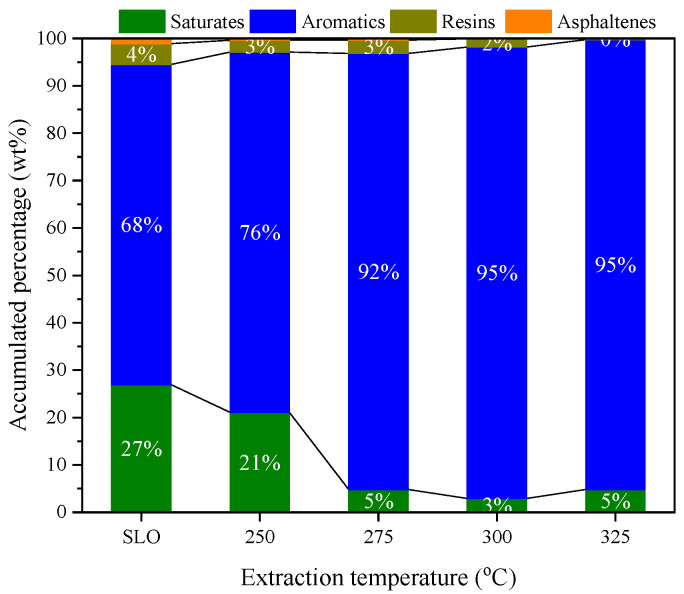
SARA distribution of SLO and extracts.

Figure 4 presents the SARA distribution of the raw SLO and the extracts obtained at different extraction temperatures. The feedstock primarily consists of saturates (27.0 wt%) and aromatics (67.6 wt%), with minor contributions from resins and asphaltenes. At 250 °C, the extract retains a composition similar to that of the original SLO, and the extraction rate of aromatics is limited to only 1.3%, indicating minimal selectivity under these conditions. However, as the extraction temperature increases, a distinct trend emerges: the proportion of aromatics in the extract steadily increases, while the contents of saturates, resins, and asphaltenes decline markedly. At 275 °C, the aromatic content rises to 92 wt%, and at 325 °C, it reaches a maximum of 95 wt%, with the corresponding aromatic extraction rate climbing to 22.6%. In contrast, asphaltenes become undetectable in the extract at elevated temperatures, and the resin fraction diminishes to below 2 wt%.

These results clearly demonstrate that Sub-CW preferentially extracts aromatic hydrocarbons from SLO, and that this selectivity becomes more pronounced at higher temperatures. The disappearance of asphaltenes and the substantial reduction in saturates suggest that polar and high-molecular-weight species are poorly soluble in Sub-CW, especially as its polarity decreases with increasing temperature. This temperature-enhanced aromatic selectivity is consistent with the observed boiling point distributions.

**Figure 5:**
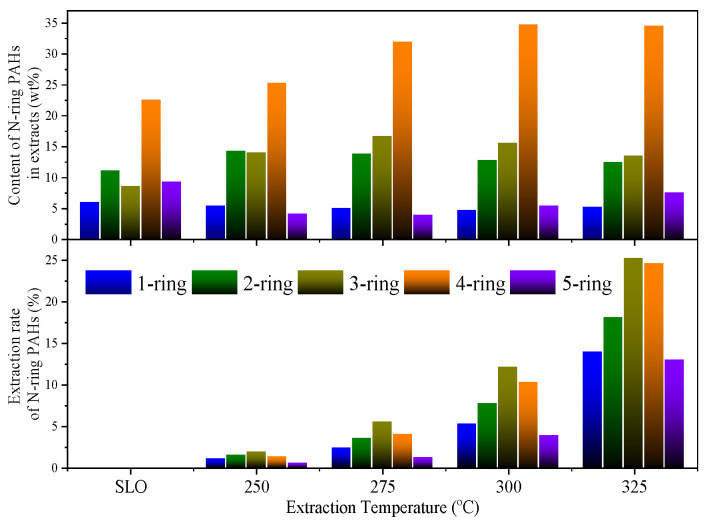
Content and extraction rate of N-ring PAHs in extracts.

Figure 5 illustrates the content and extraction rate of polycyclic aromatic hydrocarbons (PAHs) with different ring numbers in the extracts, as identified by GC-MS. In the original SLO, 4-ring PAHs dominate (22.6 wt%), followed by 2-ring (11.1 wt%) and 3-ring (8.6 wt%) species, while the contents of 1-ring and 5-ring PAHs are comparatively lower (6.0 wt% and 9.3 wt%, respectively). As the extraction temperature increases, the content of smaller PAHs (1–2 rings) in the extracts remains relatively stable, whereas the proportion of larger PAHs (3–5 rings), particularly 4-ring compounds, shows a significant and continuous increase. At 325 °C, the content of 4-ring PAHs in the extract reaches 34.6 wt%, far exceeding their original proportion in the feedstock. This reflects the enhanced solubilization of higher molecular weight, mid-polarity aromatic structures at elevated temperatures.

Consistent with this trend, the extraction rates of 2-, 3-, and 4-ring PAHs rise markedly with temperature. At 250 °C, their extraction rates are relatively low (1.5–1.9%), but at 325 °C, they increase to 18.1%, 25.2%, and 24.6%, respectively. These observations confirm that Sub-CW, under elevated temperature conditions, exhibits strong selectivity toward multi-ring PAHs—compounds that are less polar and structurally compact—due to enhanced polarity matching and reduced hydrogen bonding of the solvent phase.

**Figure 6:**
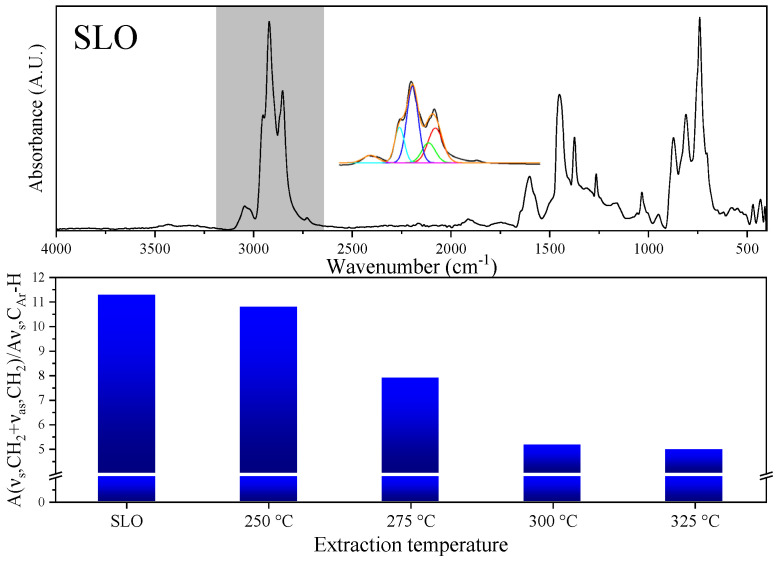
FT−IR spectrum of SLO and R_A_ of extracts obtained at different temperatures.

According to the SARA data shown in Figure 4, one may propose that by adjusting the thermodynamic state of water, effective separation of saturates and aromatics can be achieved during the SLO extraction process. The above proposal can be further confirmed by the characterization with FT-IR and ^1^H-NMR. Figure 6 shows the FT-IR spectrum of SLO, while the relevant spectra for the extracts are shown in Figure S1. The characteristic peaks listed in Table S2 can be observed in the FT-IR spectra of SLO and the extracts, and deconvolution was performed for the characteristic peaks in the range of 2600 cm^−1^–3150 cm^−1^ [30,31]. The ratio of the peak areas at 2850/2917 cm^−1^ (A_νs-CH2 + νas-CH2_) and 3040 cm^−1^ (A_νs-CAr-H_) was defined as R_A_, with the calculated R_A_ values of SLO and the extracts also shown in Figure 6. For SLO, the R_A_ value is 11.3. With the increase in extraction temperature, R_A_ decreases monotonically from 10.8 at 250 °C to 4.9 at 325 °C. A_νs-CH2 + νas-CH2_ is related to the content of alkanes and cycloalkanes, while A_νs-CAr-H_ is related to the content of aromatic hydrocarbons. Therefore, the increase in extraction temperature leads to a decrease in the content of saturates and an increase in the content of aromatics in the extract.

**Figure 7:**
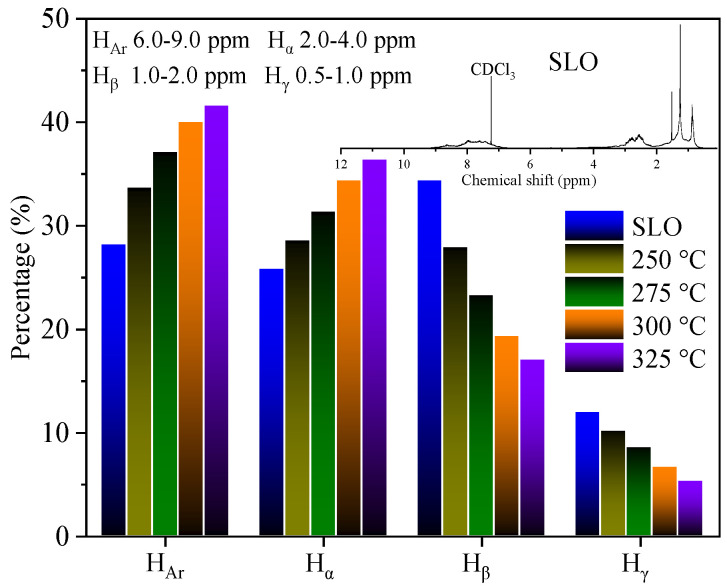
^1^H−NMR spectrum of SLO and distribution of protons in SLO and extracts.

Figure 7 presents the distribution of different proton types in SLO and the extracts obtained at various temperatures, based on ^1^H-NMR spectra. The proton signals are categorized as H_Ar_, H_α_, H_β,_ and H_γ_. In the original SLO, H_β_ dominates at 34.3%, followed by H_Ar_ (28.1%) and H_α_ (25.8%). As the extraction temperature increases, the content of H_Ar_ and H_α_ in the extract increases markedly, while H_β_ and H_γ_ decrease. At 325 °C, H_Ar_ reaches 41.6%, and H_α_ rises to 36.3%, indicating an enrichment of aromatic structures and alkyl groups adjacent to aromatic rings. In contrast, the content of H_β_ drops to 17.0%, and H_γ_ to 5.2%, reflecting the depletion of long-chain aliphatic structures in the extract.

The ratio of H_Ar_ to H_β_, often used as a semi-quantitative indicator of aromatic-to-saturate content, increases from 1.2 at 250 °C to 2.5 at 325 °C. This progressive shift reinforces the conclusion that Sub-CW selectively dissolves aromatic-rich components, especially under elevated temperature conditions. These results are in strong agreement with the GC-MS and SARA data, providing consistent spectroscopic evidence that Sub-CW extraction favors multi-ring PAHs and other condensed aromatics over saturates.

### 3.3. Solubility of Hydrocarbons in Sub-CW in Terms of HSP Distance

**Figure 8:**
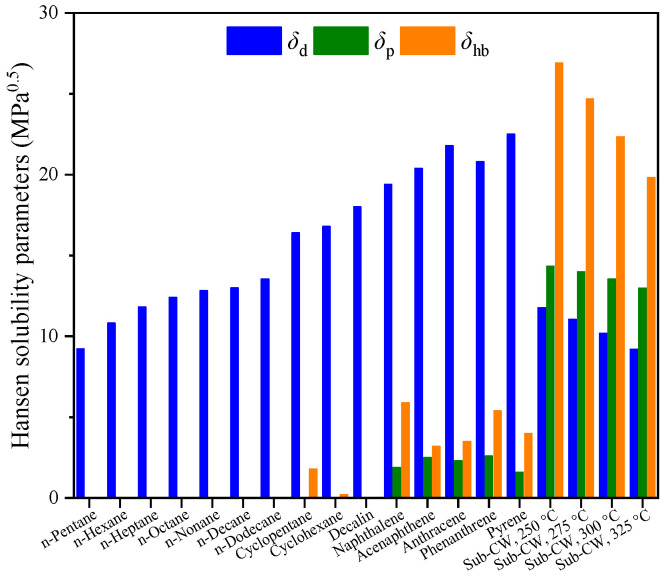
HSPs of hydrocarbons and Sub-CW.

HSP method is often applied to predict the performance of extraction systems, and the HSP distance (*R*_HSP_) is used as a quantitative basis for selecting extractants [32,33,34,35]. Figure 8 shows the literature HSP values of *δ*_d_, *δ*_p,_ and *δ*_hb_ for C_5_ to C_12_ alkanes, C_5_ and C_6_ cycloalkanes, and 1- to 4-ring PAHs [36,37,38,39]. According to the correlation recommended by the literature, the HSPs of Sub-CW in different thermodynamic states were also calculated [40].

Among the HSPs of alkanes, only *δ*_d_ is a non-zero value. As the carbon number increases from 5 to 12, *δ*_d_ increases from 9.2 MPa^0.5^ to 13.5 MPa^0.5^. The *δ*_d_ of cycloalkanes is greater than that of alkanes with the same carbon number, reaching about 16 MPa^0.5^. The *δ*_p_ of cycloalkanes is zero, and *δ*_hb_ is slightly greater than zero. Since alkanes and cycloalkanes are both saturated compounds, the dispersion force determines the HSP distribution characteristics of saturates. Due to the interaction between π electrons and special hydrogen bonds between aromatic segments, the *δ*_p_ of PAHs varies between 1.6 MPa^0.5^ and 3.6 MPa^0.5^, and *δ*_hb_ varies between 3.2 MPa^0.5^ and 5.9 MPa^0.5^ [41]. The *δ*_d_ of PAHs depends on the size of the aromatic hydrocarbons. The larger the PAH size, the larger the *δ*_d_.

The HSPs of Sub-CW can be ranked as *δ*_hb_ >> *δ*_p_ > *δ*_d_. With the increase in temperature, the HSPs of Sub-CW all decrease. Since the hydrogen bonding degree between water molecules is a sensitive function of temperature, the partial destruction of hydrogen bonds makes *δ*_hb_ respond most sensitively to temperature changes [42]. When the temperature increases from 250 °C to 325 °C, the *δ*_hb_ of Sub-CW decreases from 26.9 MPa^0.5^ to 19.8 MPa^0.5^.

**Figure 9:**
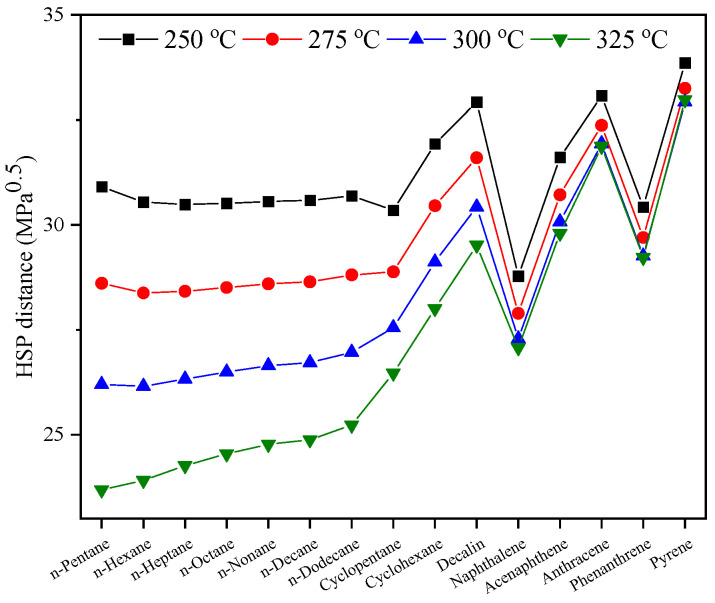
HSP distance between hydrocarbons and Sub-CW.

The *R*_HSP_ between hydrocarbons and Sub-CW was calculated based on the data shown in Figure 8, and the results are shown in Figure 9. With the increase in extraction temperature, the *R*_HSP_ between Sub-CW and various hydrocarbons is generally shortened, which is beneficial to the improvement of the yield of the extract. Unexpectedly, the *R*_HSP_ between saturates and Sub-CW is smaller than the corresponding value between aromatics and Sub-CW. According to traditional HSP theory, Sub-CW should preferentially extract saturates rather than aromatics from SLO, which is inconsistent with the experimental results. The occurrence of the above phenomenon might be related to the widely different HSP distributions of hydrocarbons and Sub-CW.

### 3.4. Modified HSP for SLO/Sub-CW Extraction System

**Figure 10:**
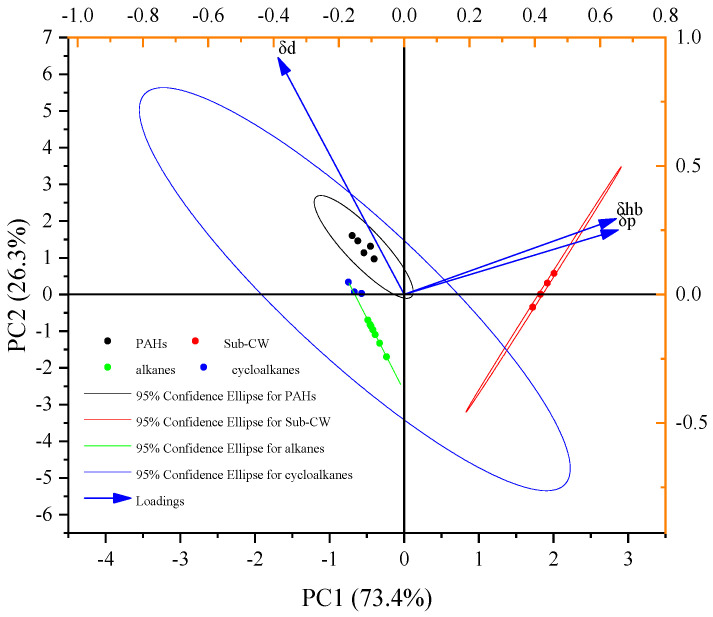
PCA of HSPs of hydrocarbons and Sub-CW.

Considering that the HSP method has a solid theoretical basis, PCA was applied to the HSPs of hydrocarbons and Sub-CW, with the results shown in Figure 10 [43,44,45].

After PCA processing, HSPs of *δ*_d_, *δ*_p,_ and *δ*_hb_ are projected into a two-dimensional space consisting of principal component 1 (PC_1_) and principal component 2 (PC_2_). 73.4% of the variance of the HSPs is retained in the PC_1_ direction, while 26.3% of the variance of the HSPs is retained in the PC_2_ direction. The HSPs of alkanes, cycloalkanes, PAHs, and Sub-CW are projected in different quadrants, indicating that there are differences in the properties of these substances. The sum of the variances retained on the two principal components reaches 99.7%, which means the data after dimensionality reduction fully reflects the differences in properties between hydrocarbons and Sub-CW. Therefore, the HSP_M_, which is dimensionality-reduced HSPs, is developed as

(3) $${HSP}_{M}=\sum_{i=1}^{n}{PC}_{i}\times {\%Var}_{i}$$

where *i* represents the number of principal components. PC_i_ denotes the projection of the HSPs onto principal component *i*, and %Var_i_ indicates the percentage of variance retained by principal component *i*.

**Figure 11:**
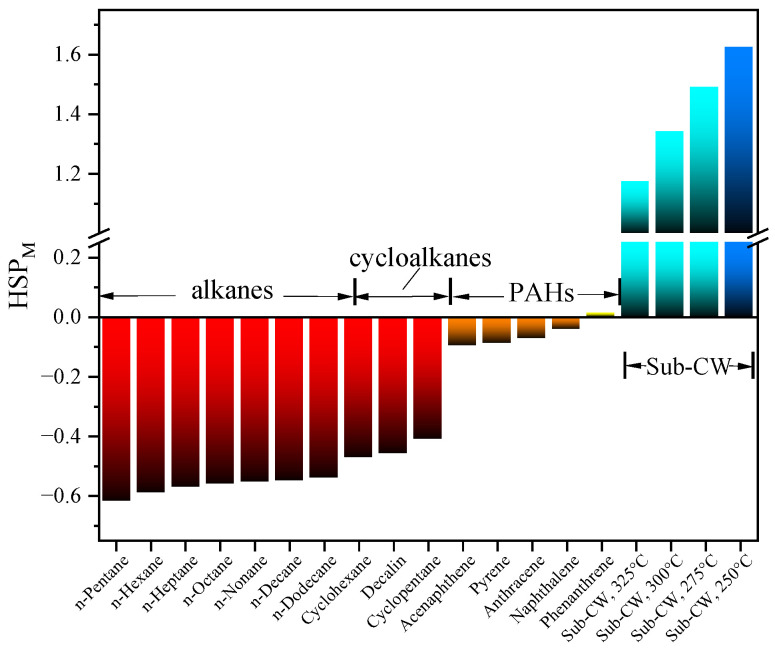
HSP_M_ of hydrocarbons and Sub-CW.

The calculated HSP_M_ of hydrocarbons and Sub-CW are shown in Figure 11. Alkanes, cycloalkanes, and PAHs are distributed in different regions according to HSP_M_, ranging from −0.62 to −0.54, −0.47 to −0.41, and −0.10 to 0.01, respectively. The HSP_M_ of Sub-CW is significantly higher than the corresponding values of hydrocarbons, ranging from 1.17 to 1.63. According to the principle of “like dissolves like”, substances with close HSP_M_ should have better mutual solubility. Therefore, the hydrocarbons preferentially extracted by Sub-CW can be ranked as: PAHs, cycloalkanes, and alkanes. The ranking based on HSP_M_ agrees well with the observed experimental results.

As the extraction temperature increases from 250 °C to 325 °C, the HSP_M_ of Sub-CW decreases monotonically, which can be attributed to the significant decrease in *δ*_hb_. Sub-CW at 325 °C has the lowest HSP_M_ of 1.17. The closer the HSP_M_ is to that of hydrocarbons, the better the mutual solubility and the higher the extraction rate. Accordingly, the highest extraction rate, as shown in Figure 2, is obtained at 325 °C.

## 4. Conclusions

Subcritical water demonstrates significant potential as an environmentally friendly and tunable solvent for the extraction of oil components from SLO. The extraction yield is strongly influenced by both temperature and water-to-oil ratio, with a maximum yield of 16% achieved at a ratio of 6:1 and 325 °C. The extracted fraction primarily consists of components with boiling points below 500 °C, among which aromatic hydrocarbons account for more than 90 wt%. These aromatics are predominantly composed of 2–4 ring PAHs. Analytical characterization by FT-IR and ^1^H-NMR confirms the compositional trends observed via SARA fractionation, collectively indicating that tuning the thermodynamic state of Sub-CW facilitates the effective separation of saturates and aromatics. By applying PCA to the HSPs of hydrocarbon components and Sub-CW, an HSP-based model, HSP_M_, was developed. This model captures the effect of Sub-CW property variation on extraction performance and yields predictions consistent with experimental trends.

It should be noted that these conclusions are derived specifically from SLO feedstock. While the developed methodology shows promise, its general applicability to other heavy oil resources, such as vacuum residue, atmospheric residue, or bitumen, requires further experimental validation. Future work will focus on extending this approach to a broader range of feedstocks to evaluate the robustness and transferability of the established correlations.

## Figures and Tables

**Table 1:** SLO applied in extraction by Sub-CW.

Density (kg/m^3^)	Dynamic Viscosity (Pa.s)	Carbon Residual (wt%)	H/C	S (wt%)	N (wt%)	Ni (ppm)	V (ppm)
1082.6	51.17	15.36	1.02	1.67	0.19	2.38	4.25
SARA Fractions (wt%)					Boiling Point Distributions		
Saturates	Aromatics	Resins	Asphaltenes		IBP~500 °C		>500 °C
27.0	67.6	4.4	1.0		58.4		41.6

## Data Availability

Data are contained within the article and Supplementary Materials.

## Supplementary Materials

The following supporting information can be downloaded at https://www.mdpi.com/article/10.3390/molecules30092079/s1. Table S1: Properties of subcritical water under experimental conditions (values calculated at saturation pressure + 0.5 MPa based on IAPWS-95 formulation); Table S2: Characteristic peaks observed in FT-IR spectra of SLO and extracts; Figure S1: FT-IR spectra of SLO and extracts obtained at different temperatures; Figure S2: ^1^H-NMR spectra of SLO and extracts obtained at different temperatures.
