# Supplementary material for: Selective Extraction of Aromatics from Slurry Oil with Subcritical Water
Source: Molecules. 2025 May 7;30(9):2079. doi: 10.3390/molecules30092079 (PMC12074435; doi:10.3390/molecules30092079)
Supplement: Supplementary file 1 [file molecules-30-02079-s001.zip › molecules-3599866-supplementary.pdf]

Supplementary Materials

Table S1. Properties of subcritical water under experimental conditions (Values calculated at saturation pressure + 0.5 MPa based on IAPWS-95 Formulation)

Temperature (°C)	Density (kg/m³)	Dielectric constant (ε)	Viscosity (μ, μPa·s)	Surface tension (mN/m)
250	800	27	106	26
275	760	24	96	20
300	713	20	86	14
325	656	17	77	9

Table S2. Characteristic peaks observed in FT-IR spectra of SLO and extracts.

Wave number (cm^{-1})	Characteristic vibrations
2850, 2917	Symmetric stretching vibration and asymmetric stretching vibration of CH_2
2870, 2955	Symmetric stretching vibration and asymmetric stretching vibration of CH_3
3040	Stretching vibration of aromatic compound C-H
1605	Aromatic conjugated C=C skeletal vibration
870	Out-of-plane bending vibration of aromatic C-H
1372	Bending vibration of CH_3
741, 809	Fingerprint region of aromatic ring

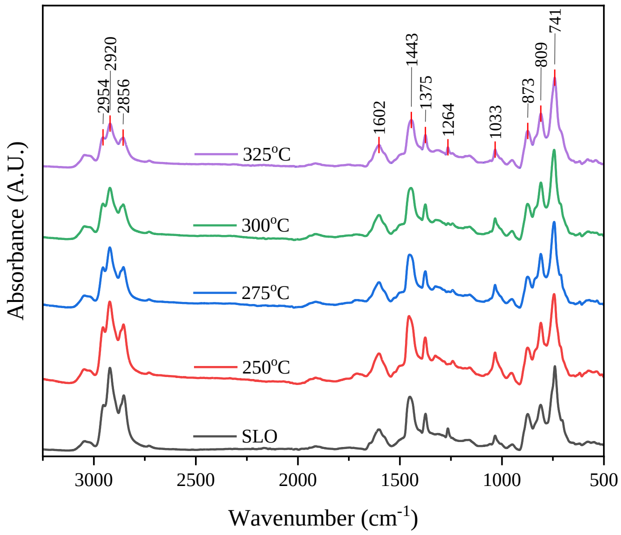

Figure S1. FT-IR spectra of SLO and extracts obtained at different temperatures.

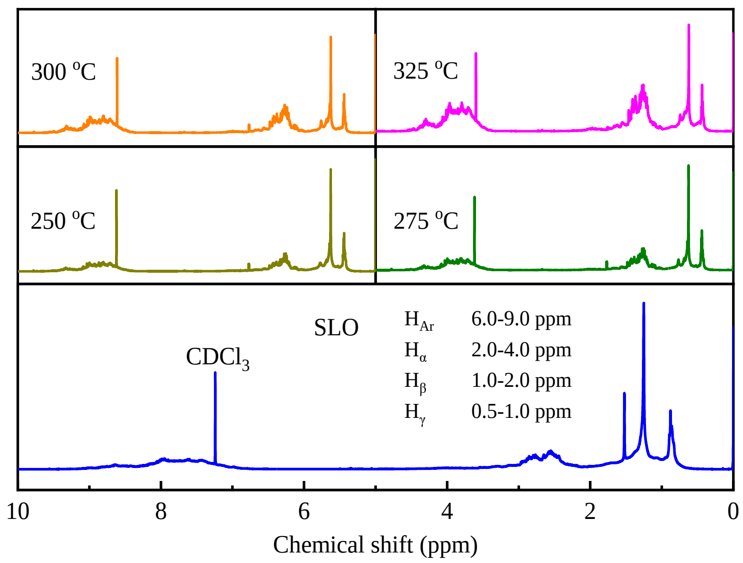

Figure S2. ^{1}H-NMR spectra of SLO and extracts obtained at different temperatures.
